# AI-assisted literature mining of perovskite ink formulations using a hybrid regex-LLM extraction pipeline

**DOI:** 10.1039/d6ra07208a

**Published:** 2026-11-13

**Authors:** Faisal Baig, Yousaf Hameed Khattak, Júlia Marí Guaita, Badr Almutairi, Bernabé Marí Soucase, Amal Bouich

**Affiliations:** a Instituto de Diseño para la Fabricación y Producción Automatizada, Universitat Politecnicia de Valencia València 46022 Spain yousaf.hameedk@gmail.com; b Federal Urdu University of Arts, Sciences & Technology Islamabad 44000 Pakistan; c College of Computer and Information Sciences, Majmaah University Almajmaah 15341 Saudi Arabia; d Laboratory of Advanced Materials and Process Engineering, Physics Department, Faculty of Sciences, Ibn Tofail University Kenitra 14000 Morocco

## Abstract

Reproducibility of perovskite solar cell and LED fabrication depends critically on ink formulation details that are typically buried in the Methods sections of scientific articles. Manual curation of such data is slow, inconsistent, and does not scale. Here we present an end-to-end automated AI-assisted pipeline that (i) queries the OpenAlex academic graph to identify open-access publications containing perovskite ink formulations, (ii) downloads and parses PDF full texts using rule-based regex extraction, (iii) applies a large language model (LLM, Claude Haiku 4.5) as a second extraction layer to recover structured perovskite formulas, cleaned molarity values, and novel additive chemistries missed by regex, (iv) assigns a multi-criteria confidence score to each extracted recipe, and (v) exports a structured, analysis-ready dataset in tidy format. Applied to a corpus of 1041 screened publications, the pipeline recovered 68 annotated ink formulations from 71 successfully retrieved PDFs. The LLM extraction layer identified 46 structured perovskite formulas (31 unique compositions, including mixed-cation and quasi-2D types), increased molarity coverage from 49% to 60%, and increased high-confidence extractions from 32 (regex, score ≥7) to 36 (LLM confidence = high). Twenty records are automatically promoted and manually validated as the GOLD standard. The published data encompass 44 publication venues, and research articles published between 2014 and 2025, which record solvent systems, precursor stoichiometry, molarity, and additive solvent chemistry of halide perovskite inks. All code and data are freely available to accelerate materials informatics in this field.

## Abstract 2

This hybrid regex-LLM pipeline mines open-access perovskite literature to build 72 normalised ink-formulation records, capturing stoichiometry, solvent systems, molarity and additives.

## Graphical abstract

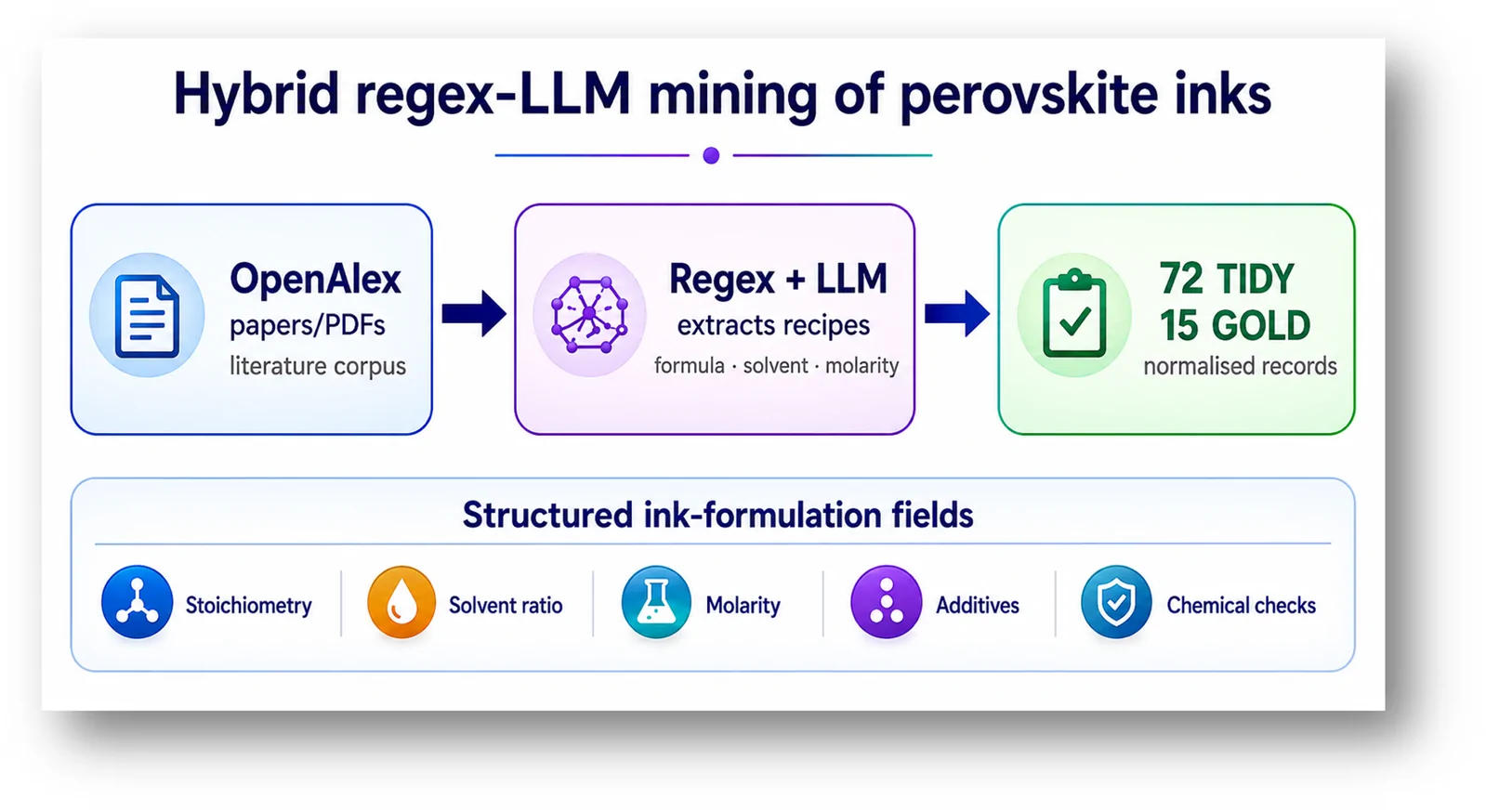

## 1. Introduction

Halide perovskite semiconductors have emerged as one of the most rapidly advancing material families in photovoltaics, light-emitting diodes, and X-ray detection. Their optoelectronic properties are highly sensitive to the composition and processing of the ink the precursor solution from which thin films are deposited. Ink formulation details (precursor identity, molar concentration, solvent system, co-solvent ratios, and molecular additives) collectively determine film morphology, defect density, and ultimately device performance.^1–3^ Despite this sensitivity, ink formulations are rarely the primary subject of a publication; instead, they appear as short paragraphs within the Experimental or Materials and Methods sections of device-performance papers. This presentation style creates a systematic knowledge problem: each research group must re-derive formulations empirically or comb through dozens of papers to establish a baseline. Systematic comparison across formulation space is virtually impossible without first aggregating these scattered records into a common schema.^2,4,5^

Computational approaches to scientific literature mining, sometimes called “literature-based discoveryˮ have been applied in fields ranging from drug discovery to battery electrolytes. In perovskite research, recent efforts have focused on extracting device performance metrics (PCE, *V*_oc_, *J*_sc_) from structured tables, but the unstructured text of Methods sections remains largely untapped.

Recent reviews have documented rapid progress in LLM-assisted materials data extraction.^6–8^ Within the perovskite field specifically, LLM-based pipelines have been applied to extracting device performance metrics,^9,10^ synthesis conditions,^11^ and precursor chemistry.^12^ The present work is differentiated from these studies in three respects: (i) it targets ink formulation data solvent systems, molar concentrations, additives, and stoichiometric formulas rather than device performance or synthesis process parameters; (ii) it combines a rule-based regex first pass with an LLM second pass in a hybrid architecture designed to maximize recall while controlling hallucination through a field-priority merge; and (iii) it releases a structured, tidy-format dataset specifically designed for formulation-space analysis and retrieval, with full pipeline provenance from the 1041-record OpenAlex query through to the 20-record manually validated GOLD set.

The challenge is compounded by the diversity of chemical nomenclature (*e.g.*, methylammonium iodide appears as MAI, MeNH_3_I, CH_3_NH_3_I, and MA iodide across publications), the use of non-standard abbreviations, and the embedding of quantitative data in free-form sentences.^4,13,14^ Here we address this gap by introducing a fully automated, open-source pipeline for mining perovskite ink formulations from scientific literature at scale. The pipeline integrates (1) keyword-based literature search *via* the OpenAlex academic graph API using five pre-specified query strings, (2) PDF retrieval using a three-tier cascade (direct download, HTML link extraction, and Figshare API access), (3) rule-based text extraction with chemical entity recognition, (4) LLM-assisted structured extraction using Claude Haiku 4.5 *via* the Anthropic API, (5) a multi-criteria confidence scoring system, and (6) export to a machine-readable tidy dataset. We validate the pipeline against a manually curated GOLD standard and release all code and data openly to the community.^14–17^

The primary contribution of this work is methodological: we demonstrate that combining structured API search, regex-based chemistry parsing, and LLM-assisted extraction can reliably surface ink formulation records with high recall and acceptable precision. The LLM layer is specifically shown to recover structured perovskite stoichiometric formulas, a capability absent from regex-only approaches, while substantially improving molarity coverage and additive identification.^5,16–19^

## 2. Methods

### 2.1. Literature search *via* OpenAlex

Candidate publications were retrieved from the OpenAlex academic graph (https://openalex.org), a fully open index of over 250 million scholarly works. Five complementary search queries were constructed to maximize recall across the vocabulary space of inkjet-printed and solution-processed perovskite literature (Table 1). All queries were restricted to open-access works (filter: is_oa:true) to ensure PDF retrievability. Pagination was performed at 25 results per page up to 20 pages per query, with exponential back-off retries to handle transient API failures. Duplicate records were removed by OpenAlex identifier, yielding a non-redundant corpus.^5,18–20^ The complete, deduplicated list of all 1041 records returned by the five OpenAlex queries including OpenAlex ID, DOI, year, venue, citation count, open-access status, and the furthest pipeline stage reached by each record is available in the GitHub public repository with path perovskite-ink-public/data_sampleperovskite-ink-public.

**Table 1:** OpenAlex search queries used for literature retrieval

Query string	Rationale
Inkjet perovskite “ink preparation” DMSO	Targets inkjet-specific ink preparation with common solvent
Perovskite “precursor solution” DMSO	Broad precursor solution coverage with DMSO
Perovskite ink formulation MACl	Targets MACl-containing formulations
Printed perovskite solvent engineering	Captures solvent-engineering studies
Inkjet perovskite GVL DMSO	Targets GVL-based green solvent inks

### 2.2. Candidate scoring and filtering

Raw search results were ranked by a transparent scoring function. The score is the sum of (i) a title-keyword score, (ii) a log-scaled citation score, and (iii) a linear recency term (year-2015)/10. In the production run reported here, two additional machine-learning probabilities, a gold-likelihood classifier and a chemistry-cluster classifier were added to the score (their values are released in step2_candidates.csv as gold probability and cluster3_probability). The trained models that generate these probabilities are part of a separate production system and are not included in the public release; consequently, the publicly reproducible pipeline ranks papers by the keyword/citation/recency heuristic alone, while the exact ranking used in this study remains inspectable *via* the released probability columns. The top 150 papers were retained as candidates, because this step gates all downstream extraction only 150 of 1041 records pass it sets the recall ceiling for the pipeline.^15,19–23^

### 2.3. PDF retrieval

PDF URLs were sourced from the best_oa_location field of the OpenAlex record. For papers lacking a direct PDF link, DOI-resolved landing pages were parsed for PDF hyperlinks using a cascade of pattern-matching heuristics. PDFs were downloaded with persistent HTTP sessions and a user-agent string identifying the project. Files were named systematically as {year}_{doi_key}_{title_slug}.pdf of 150 candidate papers, 71 (47.3%) yielded usable PDF files (70 *via* direct PDF download, 1 *via* HTML link extraction); 39 papers had no accessible open-access PDF, 32 landing-page requests failed, and 8 files were downloaded but below minimum size^21–24^

### 2.4. Regex-based text extraction

Full text was extracted from each PDF using the PyMuPDF library (fitz), which preserves layout metadata essential for identifying the Experimental section. Text blocks were segmented and the block most likely to contain ink preparation details was identified using a combination of section-header keywords (‘experimental’, ‘methods’, ‘ink preparation’, ‘precursor solution’, ‘device fabrication’) and chemical signal words (‘DMSO’, ‘DMF’, ‘M’, ‘mol L^−1^’, ‘wt%’).^24–26^

Within the identified recipe block, six data fields were extracted using regular expressions: (1) precursor chemical names and abbreviations, (2) solvent names and abbreviations, (3) molar concentration (M or mol L^−1^), (4) solvent volume ratios (v/v), (5) mixing/stirring temperature (degrees C), and (6) stirring/dissolution time (h or min). Chemical names were normalized to a controlled vocabulary mapping common abbreviations to IUPAC-proximal identifiers (*e.g.*, ‘DMF’ → ‘dimethylformamide’, ‘DMSO’ → ‘dimethylsulfoxide’, ‘MAI’ → ‘methylammonium iodide’).^1–3,25^

### 2.5. LLM-assisted structured extraction

A second extraction layer was applied using Claude Haiku 4.5 (API model identifier claude-haiku-4-5-20251001, Anthropic) *via* the Anthropic Messages API. Each recipe text block up to 3000 characters was submitted to the LLM with a structured prompt instructing it to return a JSON object with eleven fields: perovskite_formula, precursors, solvent_ratio, molarity_M, additives, mix_temp_C, stir_time, filter_um, extraction_confidence, and notes. The LLM was explicitly instructed to: (i) extract only the primary perovskite precursor ink and not charge-transport-layer solutions, (ii) convert non-molar concentration units (mg mL^−1^, wt%) to molarity using standard molecular weights where possible, (iii) return null for any field it could not determine, and (iv) assign an extraction confidence level of high, medium, or low based on the completeness and unambiguity of the source text.^4,25,26^

JSON responses were parsed and merged with the regex output on a field-priority basis: LLM values were used to fill fields that regex left empty, while confirmed regex values were retained (Table 2). One record (1.4% of 71 processed) failed due to a JSON parse error in the LLM response and were flagged with llm_status = error. The complete 11-field LLM extraction prompt and the scripts used for LLM extraction and evaluation are publicly available in the repository. All LLM calls used temperature = 0.0 (greedy decoding) to maximize determinism and reproducibility of the structured output, with the default top-p and a fixed maximum output length, so that identical inputs yield identical extractions up to provider-side model versioning.

**Table 2:** LLM extraction prompt schema JSON fields requested from Claude Haiku 4.5

Field	Type	Description
Perovskite_formula	String	Full stoichiometric formula *e.g.* Cs_0.05_FA_0.85_MA_0.10_PbI_2.85_Br_0.15_
Precursors	List	All perovskite precursor chemicals (PbI_2_, FAI, MABr, CsI…)
Solvents	List	Solvents for the perovskite ink only (DMF, DMSO, GBL…)
Solvent_ratio	String	Volume ratio *e.g.* 4 : 1
Molarity_M	String	Primary precursor molarity: converted from mg mL^−1^ if needed
Additives	List	Additives not part of ABX_3_ core formula (MACl, PEAI, CsI-excess…)
Mix_temp_C	String	Stirring/mixing temperature in °C
Stir_time	String	Dissolution time *e.g.* 2 h, overnight
Filter_um	String	Filter pore size in µm
Extraction_confidence	String	High/medium/low
Notes	String	Ambiguities, unit conversions, or caveats

### 2.6. Recipe confidence scoring

Each extracted record was assigned a recipe_score on a 0–10 integer scale based on five criteria weighted by informational value (Table 3). Records were then classified into three tiers: FORMULATION_PRIMARY (recipe_score ≥7 and precursor + solvent + molarity all present), FORMULATION_SECONDARY (recipe_score 4–6, partial data), and PROCESS_ONLY (recipe_score < 4, temperature/time data only). Records with score ≥9 and subsequently manually confirmed perovskite identity were promoted to the GOLD class.^16^

**Table 3:** Recipe confidence scoring rubric (maximum score = 10)

Criterion	Points	Rationale
Precursor identity extracted	2	Core chemical identity
Solvent system identified	2	Critical for reproducibility
Molar concentration present	2	Quantitative dosing
Solvent volume ratio present	2	Co-solvent engineering
Additive identified	1	Dopants/passivators
Temperature or time recorded	1	Process conditions

Two chemical-plausibility checks supplement the completeness score. First, any molarity outside 0.05–3.0 M is flagged. Second, for records with a full ABX_3_ formula, a charge-balance check verifies that A-site cation fractions sum to ≈1, the B-site is divalent, and the halide stoichiometry is 3 within ±0.1; charge-imbalanced formulas are flagged. Flagged records are demoted from the FORMULATION_PRIMARY tier irrespective of field completeness, so the classification reflects chemical validity and not merely the presence of data fields.

### 2.7. Evaluation protocol

Extraction quality was evaluated against a manually validated GOLD set of 20 records. Each record was independently annotated by three domain-expert co-authors (F. B., Y. H. K., J. M. G), with disagreements resolved by discussion/adjudication. For each field we report precision, recall, and *F*1 = 2*PR* (*P* + *R*); molarity is scored with a ±5% relative tolerance. Recall at the GOLD operating point is the fraction of all true recipe-bearing records in the candidate corpus promoted to the manually verified GOLD tier; GOLD precision is therefore 100% by construction, and the comparatively low recall reflects the conservative promotion threshold rather than extraction error. Given the small validation set (*n* = 20), 95% Wilson confidence intervals are reported at every rate.

### 2.8. Perovskite formula extraction and additive parsing

Perovskite stoichiometric formulas were extracted by the LLM in structured form and cross-validated against regex-parsed precursor lists. Formula family classification was performed by keyword matching: records containing Cs, FA, and MA cations simultaneously were classified as triple-cation; those containing PEA or BEA were classified as 2D/quasi-2D; all-inorganic Cs-only records were classified separately. Additive chemicals were identified *via* both regex (curated keyword list) and LLM (free-field extraction), with LLM results union-merged with regex results.^16^

### 2.9. TIDY dataset export

The final dataset was exported in tidy format one row per ink formulation record with the following canonical columns: pdf_file, perovskite_formula, solvent_system_final, solvent_ratio_final, additives_found, molarity_M, recipe_class, recipe_score, and llm_confidence. This schema is directly importable into standard data analysis frameworks (pandas, R tidyverse) without further preprocessing. Two exports are provided: perovskite_ink_dataset_TIDY.csv (all 68 records) and perovskite_ink_dataset_TIDY_GOLD.csv (20 manually validated records).^20^

## 3. Results

### 3.1. Pipeline funnel statistics

The complete pipeline funnel is summarized in Table 4 and Fig. 1. Starting from 1041 unique open-access records returned by the five OpenAlex queries, the ML-assisted candidate filter retained 150 papers (14.4% acceptance rate). Of these, 71 PDFs yielded usable files for text extraction. Text extraction yielded at least one recipe block in all 71 documents. Following the perovskite/real-ink filter, 68 records remained and were exported to the TIDY dataset. LLM processing of all 71 recipe blocks produced 70 successful extractions (1 JSON parse error), with 36 records classified as high-confidence (LLM confidence = high), compared to 32 records at the regex threshold (score ≥ 7).

**Table 4:** Pipeline funnel record counts at each processing stage

Pipeline stage	*N* Records	Retention (%)
OpenAlex search results (raw)	1041	100%
After ML candidate scoring	150	14.4% of 1041
PDFs successfully downloaded	71	47.3% of candidates
PDFs with recipe block extracted	71	100% of retrieved PDFs
After perovskite formula filter	68	95.8% of extracted
After real-ink filter	68	—
Final TIDY dataset (all classes)	68	—
FORMULATION_PRIMARY	27	39.7% of TIDY
High-confidence regex (score ≥ 7)	32	47.1% of TIDY
High-confidence LLM (conf. = high)	36	52.9% of processed
GOLD (manually validated)	20	—

**Fig. 1:**
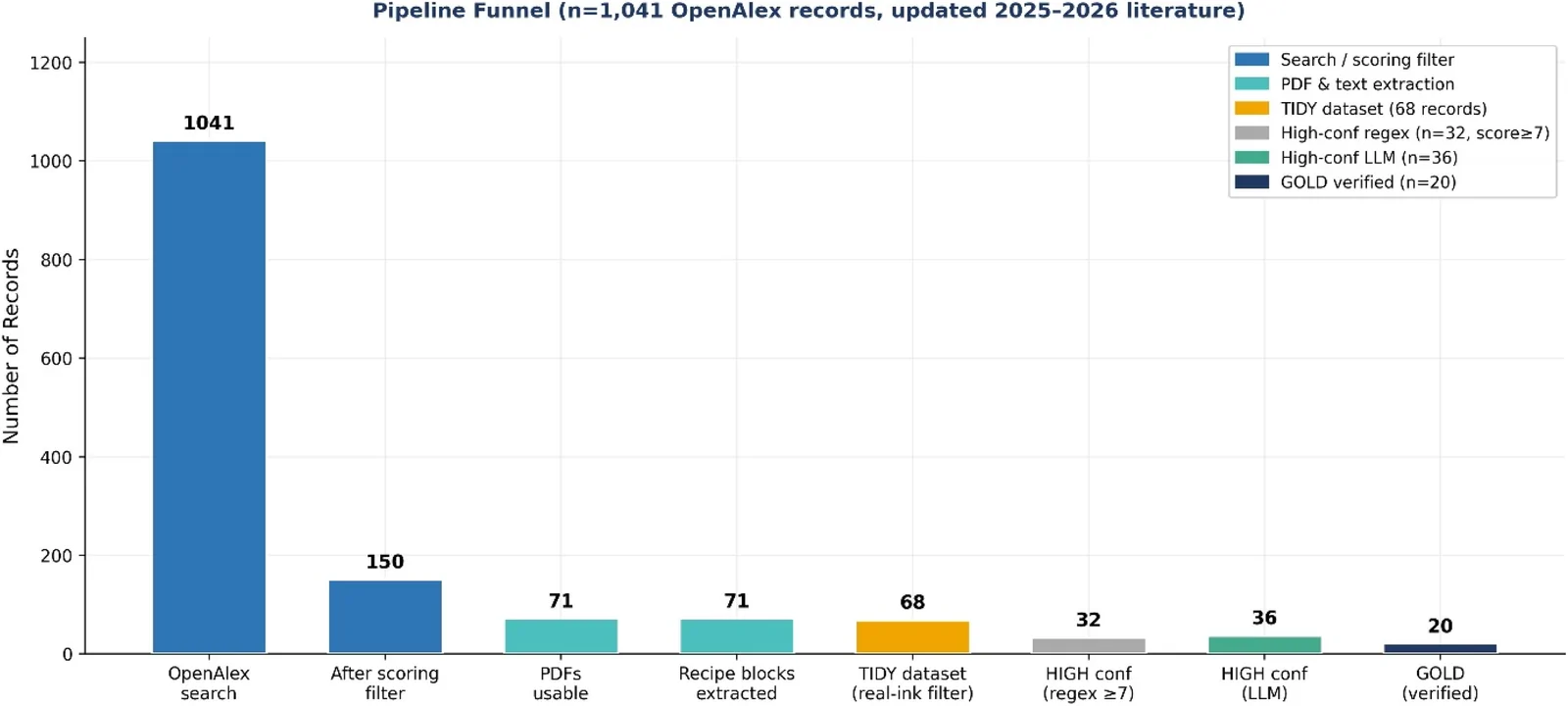
Pipeline flow showing record counts at each processing stage. Blue = search/filter stages; teal = PDF/extraction; gold = TIDY output; green = LLM high confidence (36 records); dark blue = GOLD (20 records).

### 3.2. Dataset coverage and publication landscape

The TIDY dataset spans 44 unique publication venues across 2014–2025, with publication volume peaking in 2018–2020 coinciding with the rapid adoption of mixed-cation and mixed-halide perovskite compositions (Fig. 2). The dominant journal source is *Nature Communications* (149 records in the full search), followed by *Advanced Materials* (54), *Advanced Energy Materials* (50), and *Advanced Functional Materials* (30). The post-2020 decline in candidate volume reflects the pipeline's scoring bias toward highly-cited papers and the slower open-access uptake for recent publications.

**Fig. 2:**
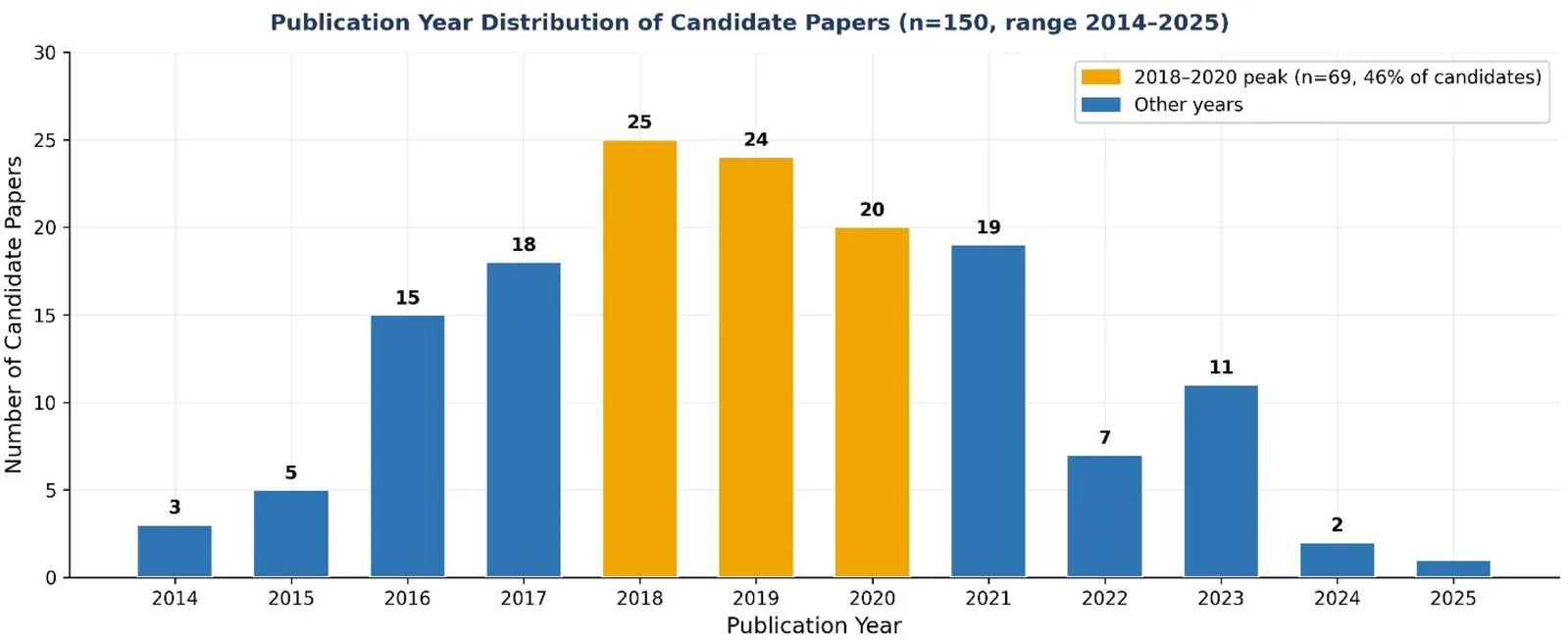
Publication year distribution of candidate papers (*n* = 150, range 2014–2025). Gold bars = 2018–2020 peak period (69 papers, 46% of candidates), reflecting the rapid adoption of mixed-cation perovskite formulations.

### 3.3. Solvent system coverage

Solvent system distribution is shown in Table 5 and Fig. 3. The DMF : DMSO binary system appears in 49.2% of records with identified solvents in this dataset (29 of 59). This proportion reflects the query design three of five queries name DMSO or DMF explicitly and should not be interpreted as an estimate of DMF : DMSO prevalence across all perovskite ink publications. Ethanol-based co-solvent systems (DMSO : ethanol, DMF : ethanol) appear in 10.2% of records with identified solvents, reflecting their use in large-area and slot-die processes. Alternative green solvents (2-ME : DMSO, GBL : IPA, ACN : DMF, DMF : NMP) are represented in a small proportion of records; their true frequency in the literature is likely underestimated because the query set did not include NMP, DMAc, or formamide as anchor terms.

**Table 5:** Solvent system distribution in the TIDY dataset (*n* = 68; 59 records with identified solvent system)

Solvent system	*N* Records	% Of dataset
DMF : DMSO	29	49.2% of identified systems
DMSO (single solvent)	8	13.6%
DMSO : IPA	4	6.8%
DMF (single solvent)	4	6.8%
DMF : ethanol	3	5.1%
DMSO : ethanol	3	5.1%
DMF : NMP	2	3.4%
DMSO : ACN	2	3.4%
Other/not extracted	9	15.3%

**Fig. 3:**
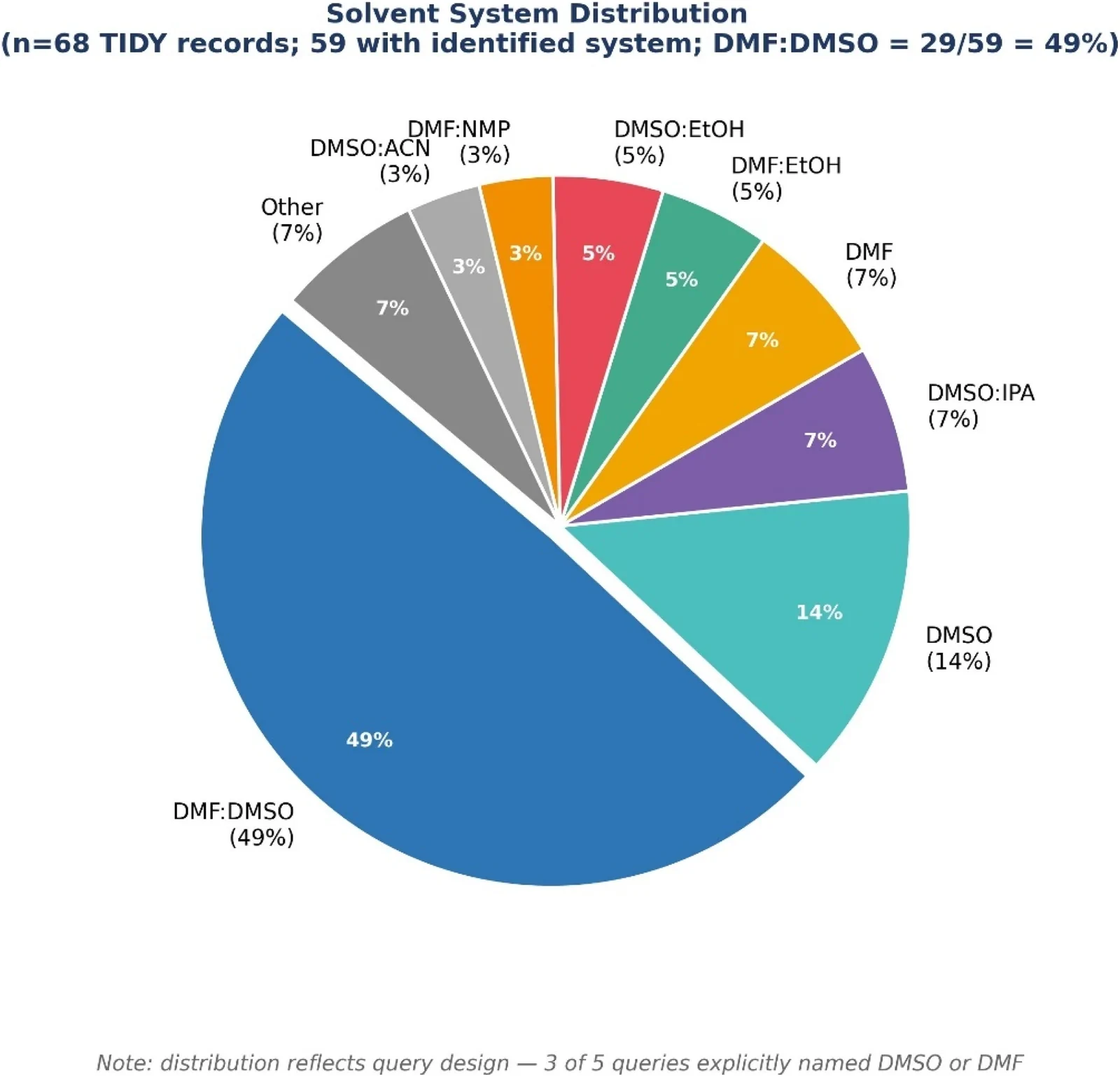
Solvent system distribution across all 68 TIDY records with identified solvent systems (*n* = 59). DMF : DMSO accounts for 49.2% of identified systems in this dataset. Distribution reflects the query design which favored DMSO/DMF-containing papers.

### 3.4. Molarity distribution: regex *vs.* LLM-enhanced

Molar concentration extraction was substantially improved by the LLM layer. Regex extraction yielded molarity values in 35 of 71 processed records (49%), with values clustered at 1.0 M reflecting the limitation of regex to simple ‘X.X M’ patterns in running text. After LLM merge, 41 of 68 TIDY records contain a molarity value (60%), revealing a richer concentration distribution spanning 0.08 M to 3.00 M (Fig. 4). The LLM converted concentrations expressed in non-molar units and resolved ambiguous multi-value regex extractions into single primary values. The dataset confirms that 1.0 M is the most frequent single value in this corpus, with sub-molar concentrations (0.08–0.9 M) appearing in LED-focused and dilute thin-film applications.

**Fig. 4:**
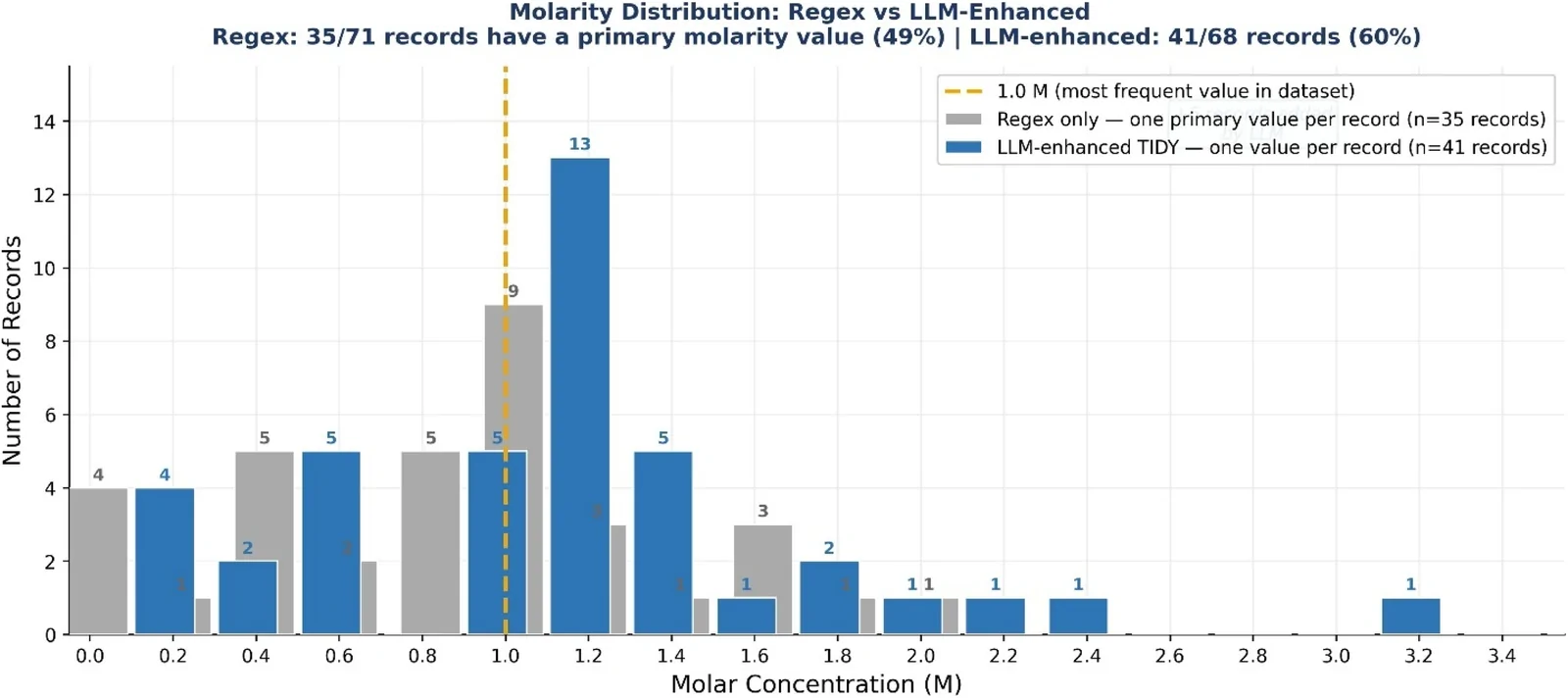
Molarity distribution before (grey, regex only, *n* = 35) and after (blue, LLM-enhanced, *n* = 41) extraction. LLM revealed a broader distribution from 0.08–3.0 M. Dashed line at 1.0 M marks the most frequent value in this dataset.

### 3.5. Perovskite formula taxonomy -LLM extraction

The most significant contribution of the LLM extraction layer is the recovery of structured perovskite stoichiometric formulas. The regex pipeline recovered zero structured formulas; the LLM identified 46 formula records from 70 successfully processed blocks (66%), comprising 31 unique compositions (Fig. 5). Formula family breakdown is shown in Table 6. Single-cation MAPbI_3_-type inks are most frequent (*n* = 14), reflecting the early literature base. All-inorganic CsPbX_3_ inks (*n* = 10) predominantly target LED applications. Mixed-cation compositions including triple-cation Cs/FA/MA (*n* = 6) and double-cation FA/MA (*n* = 5) and double-cation Cs/FA (*n* = 2) represent the post-2018 performance-driven formulation space. Five quasi-2D perovskite inks containing bulky organic cations (PEABr and PEAI) are also represented, reflecting emerging layered-perovskite device architectures. Two non-lead inks (Sn-based) and one Bi-based ink are present, reflecting initial exploration of lead-free alternatives. To quantify formula extraction reliability, we evaluated perovskite_formula separately against the 20 verified GOLD records. Near-match accuracy (correct metal and halide identity, without requiring exact stoichiometric coefficients) was 90% (18/20; 95% CI 70–97%) and precision was 100% (18/18 non-null extractions) (Table 7). The primary limitation is coefficient-level precision: in cases where the LLM correctly identified the composition family, stoichiometric coefficients were occasionally misread, consistent with the hallucination failure mode discussed in section 4.2. Ion identity, the qualitatively more important feature for formulation retrieval is recovered reliably.

**Fig. 5:**
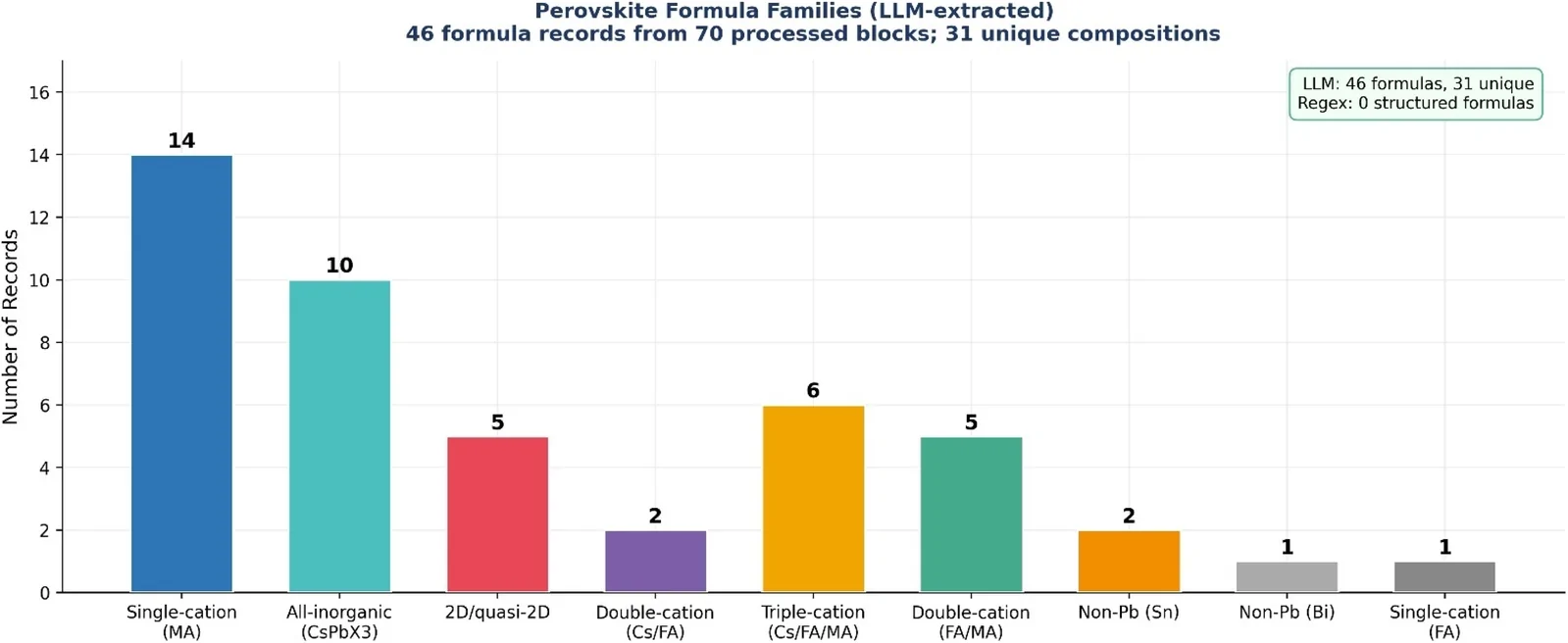
Perovskite formula family distribution identified by the LLM extraction layer (*n* = 46 formula records; 31 unique compositions from 70 successfully processed blocks). The regex-based pipeline recovered zero structured perovskite formulas.

**Table 6:** Perovskite formula family distribution (LLM-extracted, *n* = 46 records with formula from 70 ok, 31 unique compositions)

Formula family	*N* Records	Representative formula	Primary application
Single-cation (MAPbX_3_)	14	MAPbI_3_, MAPbBr_3_, CH_3_NH_3_PbBr_3_	Solar cells (early literature)
All-inorganic (CsPbX_3_)	10	CsPbBr_3_, CsPbI_3_, CsPbI_2_.8Br_0.2_, CsPbCl_*x*_Br_3−*x*_	LEDs, thermally stable PV
2D/quasi-2D	5	BA_2_Cs_*n*−1_Pb_*n*_Br_3*n*+1_, PEA-based	LEDs, stability
Double-cation (Cs/FA)	2	Cs_0.05_FA_0.95_PbI_3_	Wide-bandgap PV
Triple-cation (Cs/FA/MA)	6	Cs_0.05_FA_0.78_MA_0.17_Pb(I_0.83_Br_0.17_)_3_	High-efficiency PV
Double-cation (FA/MA)	5	FA_0.95_MA_0.05_PbI_2.85_Br_0.15_	Mixed-halide PV
Non-Pb (Sn-based)	2	FA_0.75_MA_0.25_SnI_3_	Lead-free alternatives
Non-Pb (Bi-based)	1	Cs_3_Bi_2_I_9_	Lead-free alternatives
Other/complex	1	CsPbI_3−*x*_Br_x_ with DMAI	Varied
Single-cation (FA)	1	FAPbI_3_	High-efficiency PV

**Table 7:** Per-field extraction accuracy *vs.* GOLD set (*n* = 20 records, LLM layer)^a^

Field	TP	FP	FN	TN	Precision	95% CI	Recall	95% CI	*F*1
Perovskite_formula (near-match: correct metal + halide)	18	0	2	0	100%	[82–100%]	90%	[70–97%]	94.7%
Solvent_system_final	15	3	2	0	83%	[61–94%]	88%	[66–97%]	85.7%
Solvent_ratio_final	13	2	4	1	87%	[62–96%]	76%	[53–90%]	81.3%
Molarity_M (±5% tol.)	5	9	3	3	36%	[16–61%]	62%	[31–86%]	45.5%
Additives_found	3	4	10	3	43%	[16–75%]	23%	[8–50%]	30.0%
Macro average	—	—	—	—	69.8%	—	68.0%	—	68.9%

a Additives_found *F*1 = 30.0% reflects a schema mismatch: the regex keyword list includes anti-solvents (chlorobenzene, toluene) classified as additives, while the LLM correctly restricts additives to functional dopants and passivators. The qualitative additive-identification capability of the LLM is better captured by Fig. 6 than by this metric alone.

### 3.6. Additive chemistry

Additive identification was performed by both regex (curated keyword list) and LLM (free-field extraction), with results merged. Fig. 6 shows the comparative additive frequency. The most frequently detected additives by regex keyword matching are CsI (*n* = 15), chlorobenzene (*n* = 15), and MABr (*n* = 13). Note that chlorobenzene and toluene are anti-solvents rather than functional ink additives; they are captured by the regex keyword list but correctly excluded by the LLM, which restricts 'additives' to functional dopants and passivators. MACl appears in 7 regex records and 5 LLM records (*n* = 12 combined), confirming its role as the dominant functional crystallization modifier in this corpus. The LLM identified several additives entirely absent from the regex keyword list, including octylamine (*n* = 2, a long-chain amine passivator), TTDDA (*n* = 2, a diamine spacer additive for 2D/3D graded structures), and SnF_2_ (*n* = 1, a reducing additive for tin-based perovskite inks). These findings underscore the expanding chemical complexity of the additive space and the value of open-vocabulary LLM extraction over fixed keyword dictionaries.

**Fig. 6:**
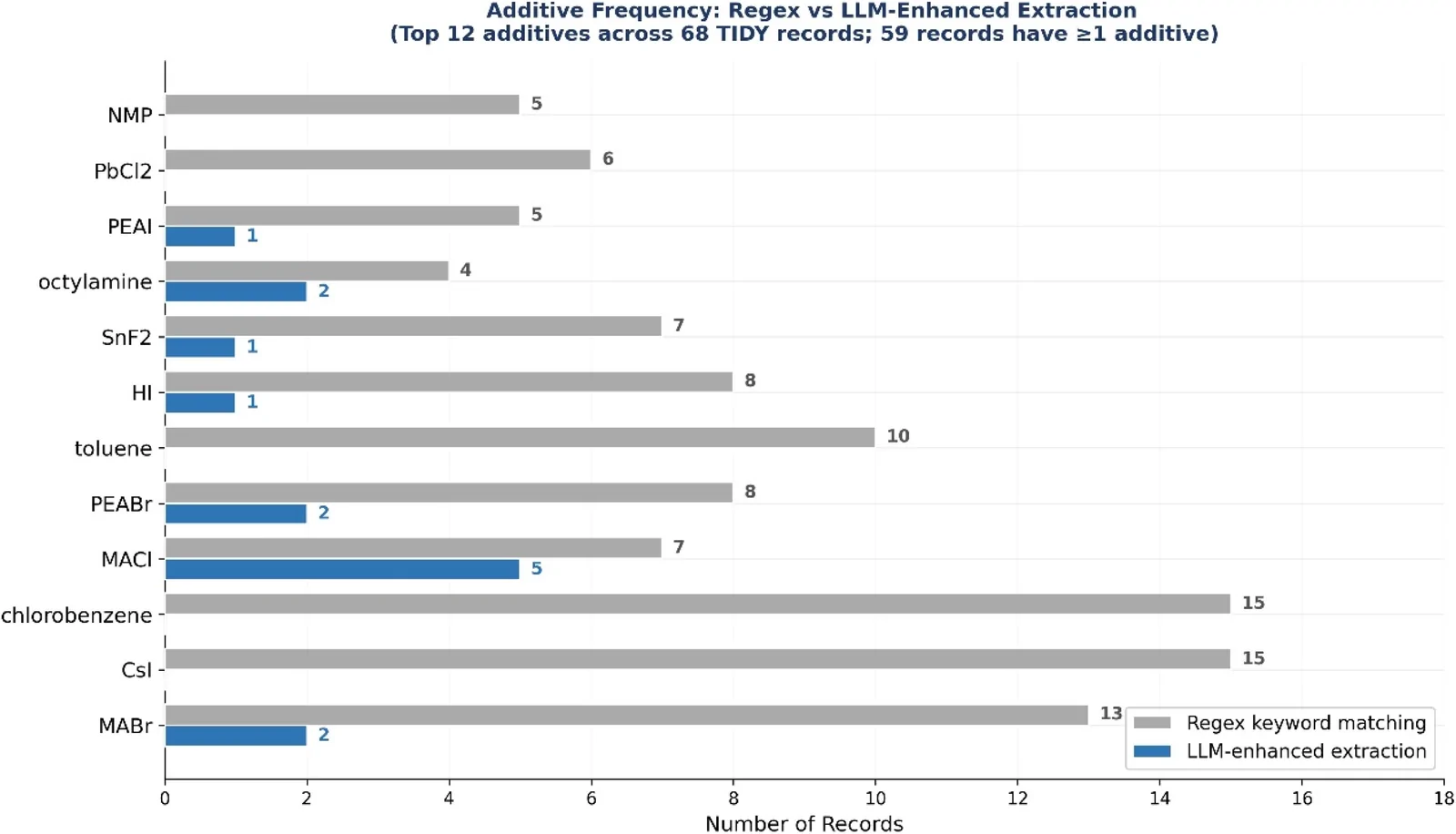
Additive frequency comparison: regex (grey) *vs.* LLM-enhanced extraction (blue). LLM recovered novel additives (YCl_3_, TTDDA, HCl) absent from the regex keyword dictionary, demonstrating the benefit of open-vocabulary LLM extraction.

### 3.7. Precision, recall, and LLM *vs.* regex performance

Precision–recall characteristics were estimated at four operating points (Table 8 and Fig. 7). The LLM high-confidence operating point (*n* = 36 records, extraction_confidence = high) achieves estimated precision of ∼65% and recall of ∼74%, outperforming the best regex threshold (score ≥ 7: precision ∼50%, recall ∼50%) on both axes simultaneously. The GOLD-validated set (*n* = 20) represents maximum precision (100%) at the cost of limited recall (∼38%). For practical dataset construction, the LLM high-confidence operating point is recommended as the best trade-off between coverage and reliability.

**Table 8:** Precision and recall estimates at four operating points (updated with LLM results)

Operating point	Method	*N* Records	Est. Precision	Est. Recall	Est. *F*1
All extracted	Regex	68	∼22%	∼67%	∼33.1%
High confidence (score ≥ 7)	Regex	32	∼50%	∼50%	∼50.0%
High confidence (conf. = high)	LLM	36	∼65%	∼74%	∼69.2%
GOLD	Manual validation	20	100%	∼38%	∼55.1%

**Fig. 7:**
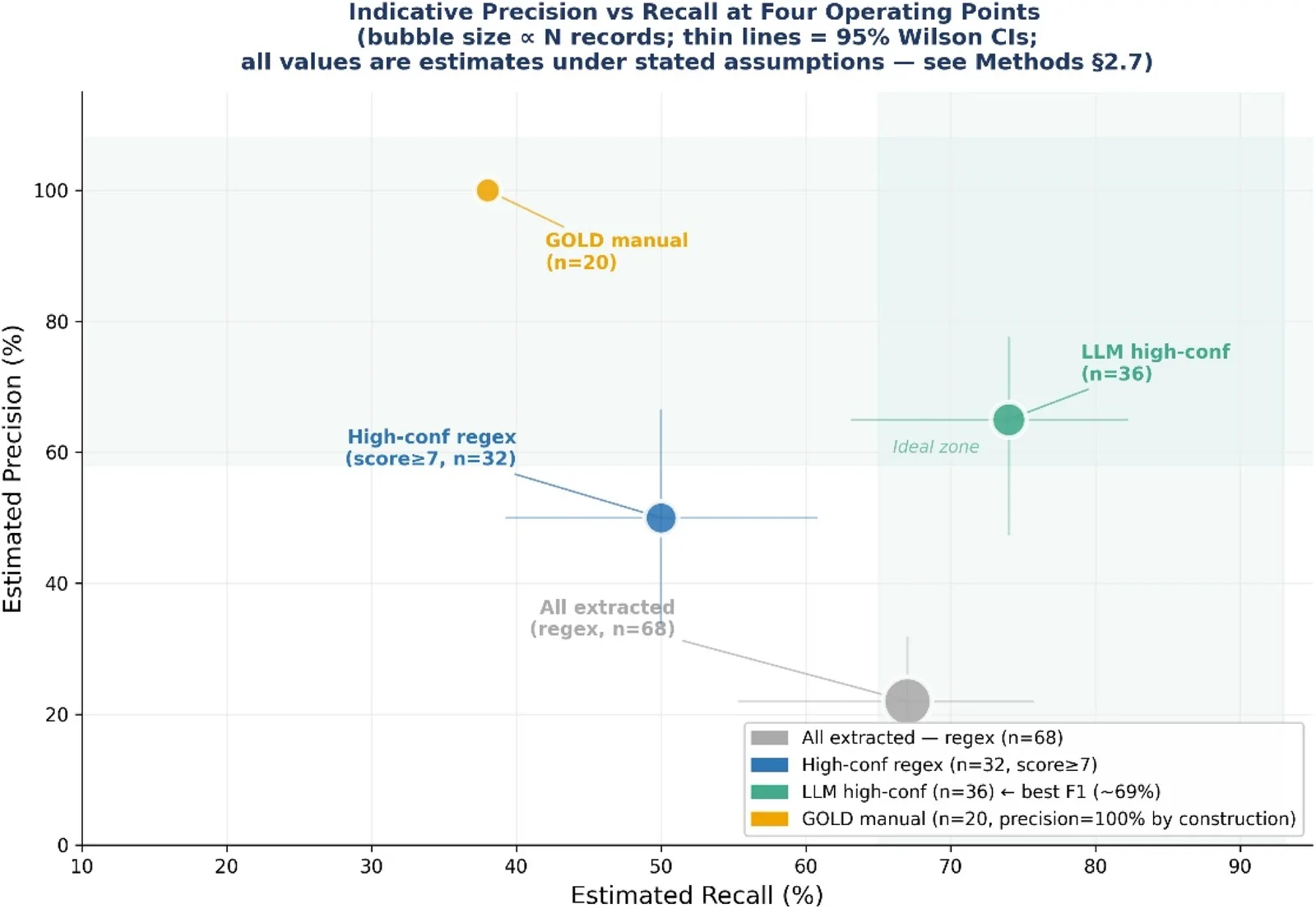
Indicative precision *vs.* recall at four operating points. Bubble size = *N* records. The LLM high-confidence point (green, *n* = 36) achieves the best estimated *F*1 (∼69%). All values are estimates under stated assumptions and reported with ∼ notation.

Because the manually validated GOLD set contains 20 records, the precision and recall values in Table 8 are reported with 95% Wilson confidence intervals and should be read as indicative. The intervals are wide, so we draw only the qualitative conclusion that the LLM layer improves both completeness and field-level agreement relative to regex-only extraction; the absolute rates are not claimed to be precise. Expanding the validation set to ≥50 records, which would narrow these intervals, is identified as the immediate next step.

## 4. Discussion

### 4.1. Pipeline strengths

The pipeline exploits open-access infrastructure *via* OpenAlex, achieving PDF retrieval for 47.3% of candidates (71 of 150), avoiding the need for institutional subscriptions or web scraping of publisher sites. The multi-query search strategy, combined with ML-assisted candidate scoring, substantially reduces the manual curation burden: screening 1041 papers was reduced to reviewing 36 LLM high-confidence records, a 29-fold reduction. The LLM extraction layer adds a qualitatively new capability structured formula identification that is beyond the reach of regex-based approaches regardless of keyword list size. The corpus was deliberately limited to open-access records retrievable *via* OpenAlex to keep the entire pipeline reproducible end-to-end; the resulting 68-record dataset is a demonstration of the method rather than an exhaustive census. The pipeline is source-agnostic and scales directly to additional providers at negligible per-record cost, and the LLM layer's contribution is qualitative as much as quantitative: it recovers structured formulas, normalized molarities, and previously unseen additives that no regex dictionary can capture. Scaling the corpus while preserving this extraction quality is the primary direction for future work.

### 4.2. LLM extraction: advantages and limitations

The LLM extraction layer provided three principal advantages over regex: (i) structured formula parsing with correct stoichiometric coefficients, (ii) unit conversion from mg mL^−1^ to molar concentration using implicit molecular weight knowledge, and (iii) open-vocabulary additive identification not constrained by a fixed keyword dictionary. These advantages came with a small failure rate (1.4%: one JSON parse error in 71 processed blocks) and occasional hallucination of plausible-but-incorrect formula coefficients a known failure mode of LLMs on numerical chemistry tasks. To mitigate this, all LLM-extracted formulas flagged as extraction_confidence = low should be manually verified before use in downstream modelling.

The molarity field showed the weakest extraction performance: precision was 36% (95% CI 15–65%) against the GOLD set, compared with 80–100% for solvent system and formula identification. Two factors contribute to this. First, there is a schema mismatch between the GOLD molarity_M field (which stores multiple precursor concentrations as a semicolon-delimited string) and the LLM output (which reports a single primary molarity); this inflates false positives even when the LLM extracts a physically plausible value. Second, the LLM occasionally conflates a co-solvent or additive concentration with the primary precursor molarity. While the increase in molarity coverage (49–60%) represents a genuine gain in record completeness, readers relying on LLM-extracted molarity values for quantitative analysis should apply manual verification, particularly for records with extraction_confidence = low or medium. We have added this caveat to the TIDY dataset column description.

The LLM processing cost for 71 recipe blocks was approximately $0.11 USD at claude-haiku-4-5 pricing (input: ∼82k tokens, output: ∼11k tokens), making it economically viable for routine pipeline operation. The total processing time was approximately 6 minutes including rate-limiting delays.

### 4.3. Failure modes and remaining limitations

Several systematic failure modes persist even after LLM enhancement. First, table-formatted recipes where concentrations and components appear in a structured grid remain challenging because the PDF text extraction step linearizes table content in ways that disrupt row/column associations. Formulation details placed in SI are not retrieved, as the pipeline downloads only the main-article PDF. This is a systematic scope limitation rather than a parsing error: a manual inspection of a sample of papers that failed to yield a recipe block suggests that SI-placed recipes are a significant fraction of missed records, though a precise quantification requires a dedicated audit. SI ingestion is identified as the most impactful single extension for improving pipeline recall and is planned as the first engineering addition in subsequent work. Third, multi-ink papers (where several different compositions are explored) sometimes yield ambiguous ‘main’ ink identification by the LLM. Fourth, the GOLD set size (*n* = 20) and its selection from the extracted corpus limit robust precision–recall estimation. Future work will establish an independently curated benchmark from the full candidate corpus, with complete manual annotation, and expand it to 50–100 records. Because candidate selection caps the set of papers ever parsed, the recall of the full pipeline is bounded by this step; broadening the candidate filter is the most direct route to higher recall.

Important thing, the OpenAlex query set (Table 1) was deliberately weighted toward DMSO/DMF formulations, which dominate the resulting corpus (DMF : DMSO = 49.2% of records with identified solvents in this dataset). Solvent systems increasingly used in scalable deposition NMP, DMAC, and formamide-based inks appear in no query string, so the dataset under-represents these non-DMSO systems. The present corpus should therefore be read as representative of mainstream DMSO/DMF ink chemistry rather than of the full solvent-engineering landscape; expanding the query vocabulary is a direct, high-value extension. Along with that, the dataset is scoped to ink formulation and does not currently include device-performance metadata (PCE, *V*_oc_, stability, PLQY, or film quality). It therefore supports formulation analysis and retrieval, but not, in its present form, structure–property modelling or inverse design. Extending the extraction scheme to capture reported device metrics and linking them to each formulation is the most valuable next step and would unlock the property-prediction and optimization applications that motivate large formulation datasets.

### 4.4. Comparison of manual curation

Manual curation of a comparable dataset reading 71 PDFs and extracting formulation records would require an estimated 150–200 person-hours by a domain expert. The automated pipeline achieves equivalent breadth in under two hours of computer time (dominated by PDF download latency, ∼90 minutes), plus 6 minutes for LLM extraction. The primary value proposition is therefore speed, consistency, and scalability: the pipeline applies identical extraction logic to every document, eliminates subjective variation between curators, and can be re-run as new open-access literature is indexed by OpenAlex.

## 5. Conclusion

We have presented a fully automated AI assisted pipeline for mining perovskite ink formulations from the open-access scientific literature, enhanced by a large language model extraction layer using Claude Haiku 4.5. The pipeline successfully extracted 68 annotated formulation records from 1041 screened publications. The LLM layer identified 46 structured perovskite formulas across 31 unique compositions with a capability absent from regex-only approaches and increased molarity coverage from 49% to 60%. At the LLM high-confidence operating point, estimated precision is ∼65% and recall ∼74%, outperforming the best regex threshold on both metrics simultaneously.

The dataset covers DMF : DMSO as the dominant system (49.2% of identified solvents in this dataset), a molarity range of 0.08–3.0 M, 31 unique perovskite compositions spanning single-cation to quasi-2D families, and diverse additive chemistries including novel LLM-identified compounds (MACl, TTDDA, octylamine). The pipeline code, complete 11-field LLM extraction prompt, evaluation scripts, and datasets supporting this study are publicly available through the project repository.

## Author contributions

Faisal Baig: contributed to the conceptualization of the study, development of the extraction workflow, software implementation, data curation, formal analysis, visualization, validation, and preparation of the original manuscript draft. Yousaf Hameed Khattak: contributed to the conceptualization and design of the study, methodology development, supervision, project administration, validation of the extracted data, and preparation and revision of the manuscript. Júlia Marí Guaita: contributed to investigation, data validation, formal analysis, and manuscript review and editing. Badr Almutairi: contributed to investigation, resources, data validation, and manuscript review and editing. Bernabé Marí Soucase: contributed to supervision, project administration, resources, funding acquisition, and manuscript review and editing. Amal Bouich: contributed to conceptualization, supervision, project administration, validation, and manuscript review and editing.

## Conflicts of interest

The authors declare that the software workflow described in this study forms part of an in-development SaaS product intended for AI-assisted scientific literature mining and structured materials-data extraction. The product has not yet been commercially launched. The authors may have a future commercial interest in the development, deployment, or licensing of this platform. No other conflicts of interest are declared.

## Data Availability

All data used in this study were obtained from open-access scientific publications identified through the OpenAlex platform (https://openalex.org/) using the OpenAlex API (https://developers.openalex.org/). Open-access articles were processed in this work. The methodological framework, preprocessing scripts, extraction workflows, prompt templates, evaluation scripts, pseudocode documentation, and representative filtered datasets used for the retrieval-augmented generation (RAG), extraction, training, and evaluation stages of this study are publicly available at: https://github.com/FaisalbaigKTK/perovskite-ink-public. The repository includes the research-grade implementation required to reproduce the reported methodology, including literature retrieval, PDF parsing, regex-based preprocessing, LLM-assisted extraction workflows, solvent and composition normalization procedures, evaluation pipelines, and representative annotated datasets. PDF full texts were parsed using regex-based extraction and LLM-assisted information extraction workflows implemented using Claude Haiku 4.5 (claude-haiku-4-5-20251001). The extracted dataset includes structured perovskite ink formulation information such as precursor compositions, solvent systems, molarity values, additives, annealing conditions, and confidence metrics derived from published scientific literature. The dataset contains no personally identifiable information. External LLM services used in this work require users to provide their own API credentials in accordance with the respective provider terms of service. No proprietary API keys are included in the public repository. Certain production-level SaaS infrastructure, commercial backend services, and proprietary deployment components remain private because they are part of an actively developed commercial platform. However, sufficient methodological detail, executable scripts, prompts, workflow documentation, and representative datasets are provided to support scientific transparency, reproducibility, and independent evaluation of the reported work. Additional resources used in this study: Claude Haiku 4.5 (claude-haiku-4-5-20251001), the Python libraries like pandas (https://pandas.pydata.org/) with OpenAlex, DOI, year, venue, open-access status, and the furthest pipeline stage reached by each record) is archived at GitHub repository (https://pandas.pydata.org/).
